# Supplementary material for: Dopamine Transporter, PhosphoSerine129 α-Synuclein and α-Synuclein Levels in Aged LRRK2 G2019S Knock-In and Knock-Out Mice
Source: Biomedicines. 2022 Apr 12;10(4):881. doi: 10.3390/biomedicines10040881 (PMC9027615; doi:10.3390/biomedicines10040881)
Supplement: Supplementary file 1 [file biomedicines-10-00881-s001.zip › biomedicines-1675345-supplementary.pdf]

Dopamine Transporter, PhosphoSerine129 α-Synuclein and α-Synuclein Levels in Aged LRRK2 G2019S Knock-In and Knock-Out Mice

Chiara Domenicale [1,#], Daniela Mercatelli [1,2,#], Federica Albanese [1], Salvatore Novello [1,3], Fabrizio Vincenzi [4], Katia Varani [4] and Michele Morari [1,*]

[1] Department of Neuroscience and Rehabilitation, University of Ferrara, 44121 Ferrara, Italy
[2] Technopole of Ferrara, LTTA Laboratory for Advanced Therapies, 44121 Ferrara, Italy
[3] Laboratory of Molecular and Chemical Biology of Neurodegeneration, Brain Mind Institute, Ecole PolytechniqueFédérale de Lausanne (EPFL), CH-1015 Lausanne, Switzerland
[4] Department of Translational Medicine, University of Ferrara, 44121 Ferrara, Italy

* Correspondence: m.morari@unife.it
[#] These Authors contributed equally to this work.

Supplementary materials

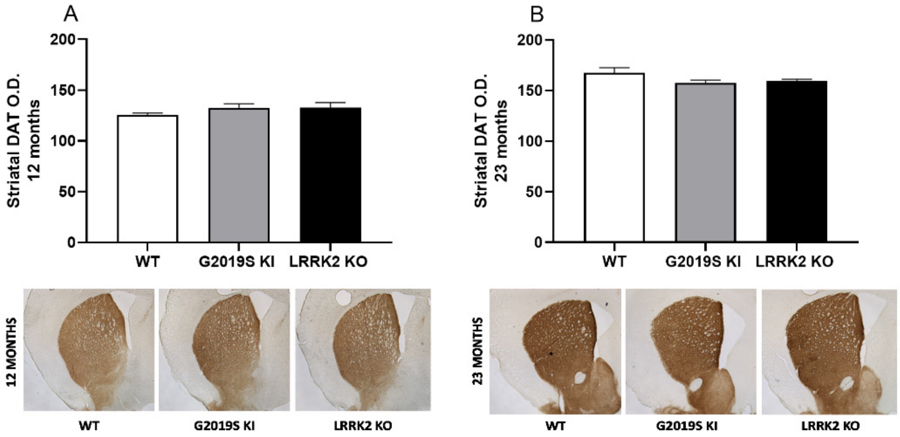

Figure S1. DAT immunoreactivity in striatal slices of 12-month-old (**A**) and 23-month-old (**B**) G2019S KI and LRRK2 KO mice in comparison with age-matched WT mice. Optical density (O.D.) of DAT immunostaining was quantified. Representative images were provided in the lower panels Data are means ± SEM of n=7–9 mice per group.
